# Trend Technologies for Robotic Fertilization Process in Row Crops

**DOI:** 10.3389/frobt.2022.808484

**Published:** 2022-04-27

**Authors:** Christyan Cruz Ulloa, Anne Krus, Antonio Barrientos, Jaime del Cerro, Constantino Valero

**Affiliations:** ^1^ Centro de Automática y Robótica, Consejo Superior de Investigaciones Científicas, Universidad Politécnica de Madrid, Madrid, Spain; ^2^ Departamento de Ingeniería Agroforestal, ETSI Agronómica, Alimentaria y de Biosistemas, Universidad Politécnica de Madrid, Madrid, Spain

**Keywords:** smart farming, robotics in agriculture, multi-spectral images, precision agriculture, point clouds

## Abstract

The development of new sensory and robotic technologies in recent years and the increase in the consumption of organic vegetables have allowed the generation of specific applications around precision agriculture seeking to satisfy market demand. This article analyzes the use and advantages of specific optical sensory systems for data acquisition and processing in precision agriculture for Robotic Fertilization process. The SUREVEG project evaluates the benefits of growing vegetables in rows, using different technological tools like sensors, embedded systems, and robots, for this purpose. A robotic platform has been developed consisting of Laser Sick AG LMS100 × 3, Multispectral, RGB sensors, and a robotic arm equipped with a fertilization system. Tests have been developed with the robotic platform in cabbage and red cabbage crops, information captured with the different sensors, allowed to reconstruct rows crops and extract information for fertilization with the robotic arm. The main advantages of each sensory have been analyzed with an quantitative comparison, based on information provided by each one; such as Normalized Difference Vegetation Index index, RGB Histograms, Point Cloud Clusters). Robot Operating System processes this information to generate trajectory planning with the robotic arm and apply the individual treatment in plants. Main results show that the vegetable characterization has been carried out with an efficiency of 93.1% using Point Cloud processing, while the vegetable detection has obtained an error of 4.6% through RGB images.

## 1 Introduction

The increase in organic vegetable consumption and the search for more sustainable and environmentally friendly agriculture have triggered factors to give rise to new technologies to optimize vegetable cultivation and treatment processes for supplying the constant market demand (Pachapur et al., 2020; Balkrishna, 2021; Lichtfouse, 2021; Linaza et al., 2021; Singh, 2021). These processes are mainly focused on the detection, irrigation, fertilization of vegetables and fruits during their growth and harvest phases (Gonzalez-de Santos et al., 2020; Tang et al., 2020; Wu et al., 2021).

Conventional fertilization systems focus on spraying vast cultivation areas using sophisticated and expensive irrigation equipment, covering even large hectares. (Abd El-Azeim et al., 2020; Mingotte et al., 2021). However, these systems do not consider the plants’ specific needs, nor are they eco-friendly with the soil, which over time can cause its degeneration and erosion. (Tripathi et al., 2020; Tudi et al., 2021; Kashyap et al., 2021; Fentahun Adametie, 2020).

The main precision agriculture applications nowadays use optical sensors such as multispectral cameras, laser-type sensors, and RGB (Red, Green, and Blue) cameras. (Qiu et al., 2019; Hemming and Rath, 2001; Puri et al., 2017; Saddik et al., 2021; Mathew et al., 2021; Tellaeche et al., 2011).

The main developments carried out with multispectral cameras are based on the vegetative analysis of plant growth, using NDVI indices, which have been shown to provide relevant information for making decisions in fertilization applications. (Paoletti et al., 2019; Cardim Ferreira Lima et al., 2020; Lu et al., 2020; Zhou et al., 2021).

On the other hand, the main applications of laser-type sensors are reconstructing vegetative environments for their analysis using clustering techniques and point cloud processing. (Krus et al., 2020; Mesas-Carrascosa et al., 2020; Cruz Ulloa et al., 2021; Schunck et al., 2021).

This work has been developed as part of the Sureveg project, which uses leading-edge technologies, such as sensors, robotic systems, and control boards with embedded processing capacity to improve row crop production. Sureveg (2020).

A robotic platform equipped with sensory systems and a robotic actuator has been implemented to validate this proof of concept. Evaluating the advantages of using these three types of sensors (RGB, Multispectral, and Laser).

The main contribution of this work focuses on qualitatively and quantitatively analyzing the use of laser sensors and multispectral and RGB cameras to influence extracting crop characteristics, obtaining relevant information (NDVI index, RGB Histograms, Point Cloud Clusters) about the status of each plant within the crop row for subsequent decision-making in a robotic fertilization process.

The tests carried out to validate the proposed method have been executed at ETSIAAB-UPM, different rows have been planted with cabbage and red cabbage. The tests have been carried out during different stages of plant growth to collect data. The main results show that the information provided by these sensors and their combination allows optimizing the selective process of vegetables that require the application of fertilizer.

This work is structured as follows: in materials and methods, Section 2, the experimental fields, and the hardware and software are introduced in detail, followed by the results in Section 3. Finally, the conclusions in Section 4 summarize the main findings.

## 2 Materials and Methods

### 2.1 Hardware and Field Test

**FIGURE 1:**
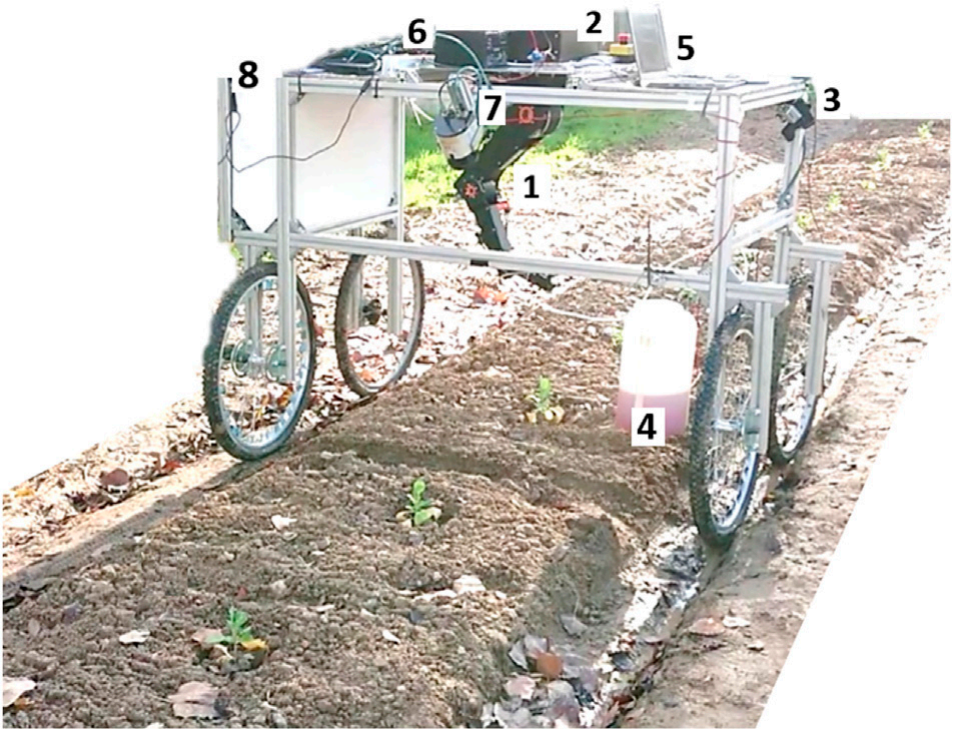
Mobile robotic platform in a cabbage row.

The different components used in this work are shown in Table 1. The components have been assembled on the platform shown in Figure 1 (platform on a row of cabbage).

**TABLE 1:** Mobile platform components.

Number	Component	Amount	Description
1	Robotic Arm Igus CPR 5DOF	1	Actuator
2	Parrot Sequoia	1	Multi-spectral camera
3	daA4200-30mci-NVJET-NVDK	1	RGB Camera
4	Fertilizer deposit, pump and nozzle	1	Fertilization system
5	PC, Jetson Nano and Jetson Xavier	3	ROS Control System
6	Control box	1	Electrical system
7	Lidar (SICK AG LMS100)	3	2D Laser Sensor
8	Wheelded Aluminum Structure	1	Mobile platform

The robotic platform (Figure 1) was assembled using Aluminum profiles (40*x*40), with four wheels to move along the crop field. The platform consists of an actuation system (Robot Igus CPR 5DOF + nozzle) and a sensory system capable of acquiring data from the cultures through a lidar system, RGB camera, and Multispectral camera (green (550 *nm*), red (660 *nm*), red edge (735 *nm*), near-infrared (790 *nm*), RGB (1,280 × 960 *pixels*)). The kinematic rig configuration is based on a four-wheel differential model.

Sensory processing has been carried out using the Jetson Xavier card and the control of actuation systems through the Jetson Nano card. The data processing and information gathering algorithms have been executed through ROS.

Field test were developed in the crops of ETSIAAB - UPM (40°26′38.9*″*N 3°44′19.3*″*W). Where rows have been grown with cabbage and red cabbage, different studies related to the Sureveg project have been previously developed focused on:

• Application of multispectral images to develop fertilization strategies and monitoring the state of vegetables in Cardim Ferreira Lima et al. (2020).
• Reconstruction and analysis of multispectral images in crop rows in Krus et al. (2021).
• Acquisition of vegetable characteristics in cultivation rows through a lidar system in Krus et al. (2020).
• The platform location system within the crop using a point cloud processing based system described in Cruz Ulloa et al. (2021).

### 2.2 Methods

**FIGURE 2:**
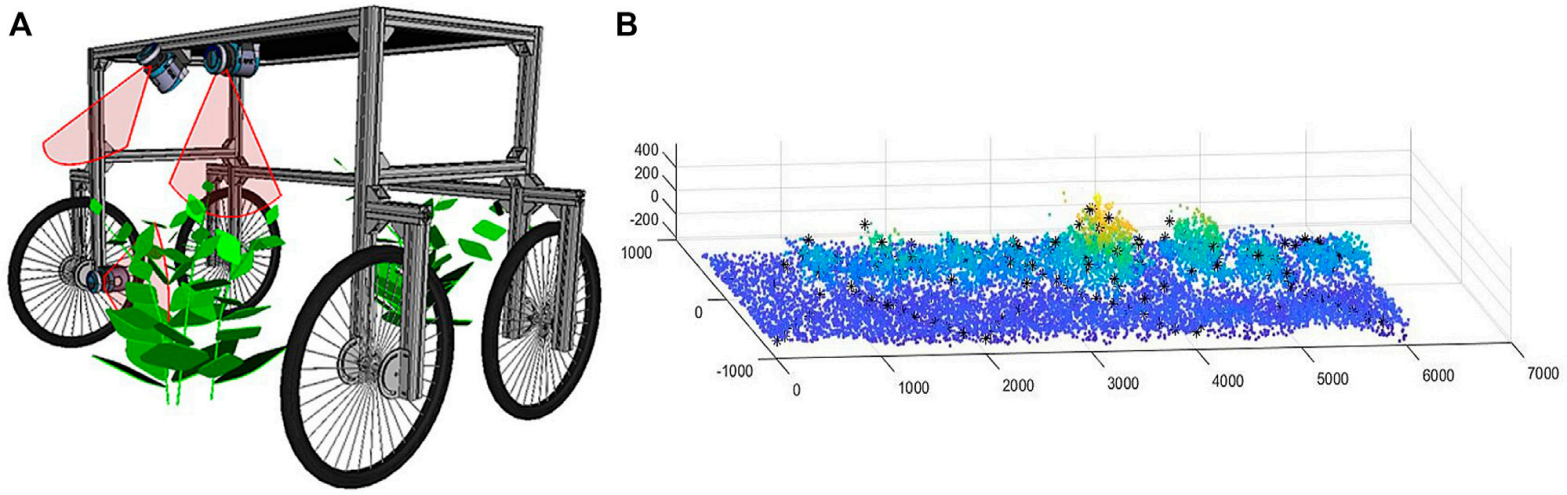
**(A)** Arrange laser sensors on the platform and coverage range for the point cloud acquisition process **(B)** Partial reconstruction of a crop row according to the advance of the robotic platform.

The different crop rows have been reconstructed using the three types of sensors: laser (point clouds), RGB camera, and Multispectral camera (mosaics). This reconstruction has developed by moving the platform along the entire row at a speed of 0.1 m/*s*. Figure 2A shows the laser sensors arrangement on the platform and coverage range for the point cloud acquisition process of a row (cabbage). The data captured by each sensor are integrated as a function platform advance, measured by an encoder. Figure 2B shows a partial section of the row crop captured.

The information captured has been processed using the strategies described below, extracting relevant information from each information source.

Lidar System fulfils is to help with the location within the environment, since although the plants do not have a complex morphology, the process of location outdoors is complex. Specially in areas with denied GPS.

#### 2.2.1 Crop Row Reconstruction and Point Cloud Analysis

**FIGURE 3:**
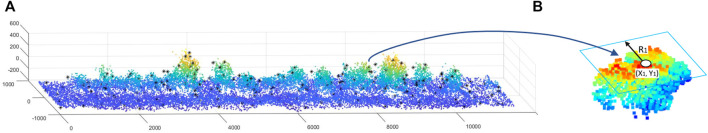
**(A)** Reconstruction of a cabbage crop row **(B)** Individual cluster of a cabbage plant.

Laser data from Sick sensors has been captured with a resolution of 1 *mm* during the advancement of the platform, supported with the use of an encoder to measure the platform advance. Figure 3A shows a reconstructed cabbage culture row using this method.

From the point cloud of the row, the individual clusters (point cloud of each floor) have been extracted. These individual clusters allow us to know their centroid characteristics, height, width, length, and location in the crop row. Figure 3B, shows one of the clusters extracted from the row.

The following iterative method has been applied to obtain the individual clusters, which comprises different stages such as:

• Soil elimination through thresholding of heights.
• Outliers extraction.
• Application of the unsupervised learning method (K-means) for clustering described according to Equation (1) (Qi et al., 2017). Segmenting the resulting data, grouping them in clusters and defining their centroids, for the subsequent treatment in the fertilization phase.

The first part of the K-means iterative method, takes the rows and assigns them to the nearest centroid based on the Euclidean square distance. The second part consists of recalculating the centroids of each group, based on each cluster mean assigned in the previous iteration.

This process is executed cyclically until it detects if there are no changes in the newly assigned groups, if several iterations are met or the minimizing sum of squared error *SSE* criterion is met, given by Equation (1).

$$SSE=\overset{k}{\underset{j=1}{\sum }}\overset{n}{\underset{i=1}{\sum }}\delta_{ij}{\left\|p_{i}-m_{j}\right\|}^{2}$$ (1)

Where, *k* is the number of clusters, *n* the clusters number of points (*j*), $\left\|i_{i}-m_{j}\right\|$ the distance between *p*_*i*_ the analyzed point and *m*_*j*_ the center of the cluster.

The method’s effectiveness has been evaluated based on the mean error obtained from the parameters obtained by each cluster’s algorithm (radius and height) concerning the values quantified with field measurement instruments (graduated pole).

#### 2.2.2 RGB Mosaic Analysis

**FIGURE 4:**
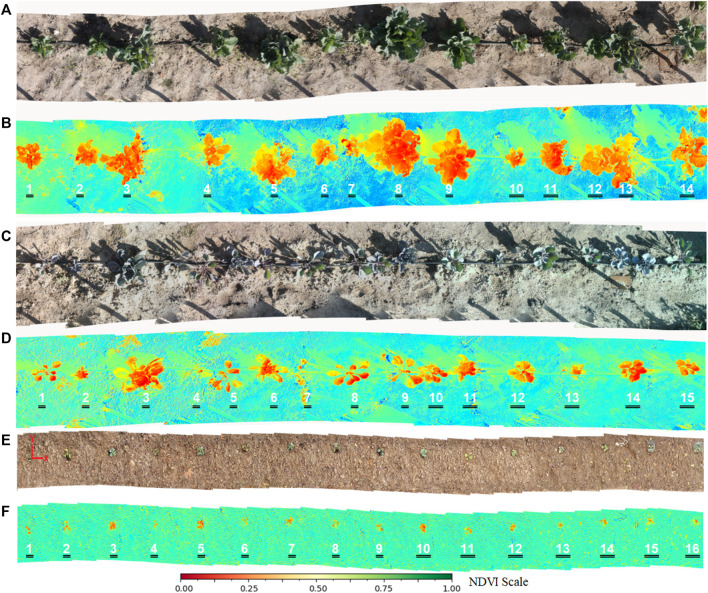
Rgb **(A,C,E)** and Multispectral **(B,D,F)** crop row mosaics.

Figure 4 shows the reconstruction of the cabbage and red cabbage rows for both RGB images (Figure 4A,C,E) and corresponding Multispectrals (Figure 4B,D,F). The methodology for RGB and Multispectral row reconstruction mosaic has been previously developed in (Krus et al., 2021).

RGB images make it possible to automatically detect the vegetables within the crop row through computer vision techniques and, above all, based on the information provided by the histograms of the Green color channel.

Depending on the extracted color channel, dynamic thresholds have been established in the histogram, considering the area that covers the highest concentration of channel information within the histogram. 70% of the histogram information has been taken around the zone of maximum concentration. Values outside this range have been considered to correspond to weeds, so they have been discarded.

Classical vision techniques have been applied for processing, such as erosion-expansion filters, thresholding, segmentation, and edge detection. Finally, a mask is obtained with the detected vegetables, applied to the original image to show the detection results. The error of the number of vegetables detected in each row, concerning the total number of vegetables counted, has been analyzed to evaluate the efficiency of the detection.

#### 2.2.3 Multispectral Analysis of Crop Rows

The multispectral images will be used to evaluate if the plants need the application of fertilizer based on the NDVI (Normalized Difference Vegetation Index) analysis. They are measuring green vegetation through a normalized ratio ranging from -1 to 1. (Paoletti et al., 2019; Moriarty et al., 2019).

This parameter is calculated based on Eq. (2) (Cardim Ferreira Lima et al., 2020). Where the indices NIR(Near-InfraRed), RED (Red Edge) are involved.

$$\text{NDVI}=\frac{NIR-RED}{NIR+RED}$$ (2)

Depending on the plants detected in the previous section, the same mask can be established (used to detect plants within the row), separating each plant from the mosaic, to obtain an average value of the NDVI index for each plant.

The value of this parameter allows defining whether or not each plant requires the application of fertilizer. Plants with values lower than the range [0.33–0.66] are considered moderately healthy. If the value is in the range [0–0.33], the application of fertilizer is required (Brown, 2015; System, 2019).

## 3 Experiments and Results

This section shows the main contributions and results from the proposed methodology applied to the data captured in the cultivation fields through different sensory sources.

### 3.1 Plants Characterization Using Point Clouds

**FIGURE 5:**
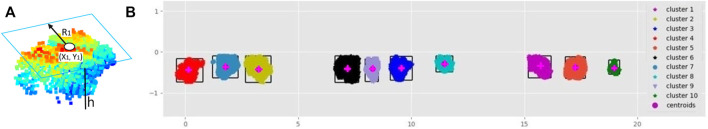
Point clouds quantification to characterize the row vegetables **(A)** View 3D, **(B)** Top View.

This subsection shows the result of applying clustering processing to a cabbage crop row. The different geometric parameters have been calculated based on an automatic algorithm process, the algorithm first part, groups the point cloud in “n” small point clouds using K-means Clustering Algorithm (the parameter “n” is defined by the user). The second part takes each cluster maximum and minimum values to define the centroid and radium for each plant. The parameter “n” is variable and depends on the plants’ number in the crop row. For Figure 5, this parameter is 10. The parameter “n” is entered as input data of the algorithm, since a criterion is required to be able to generate the clusters.

Figure 5B shows the clustering of the plants, identified by different colors from a top view, the outer edges and the centroids with a pink point have been marked with boxes. The parameters of each cluster (Centroid (*x*, *y*), Radius *R*, height *h*) are detailed in Figure 5A.

**FIGURE 6:**
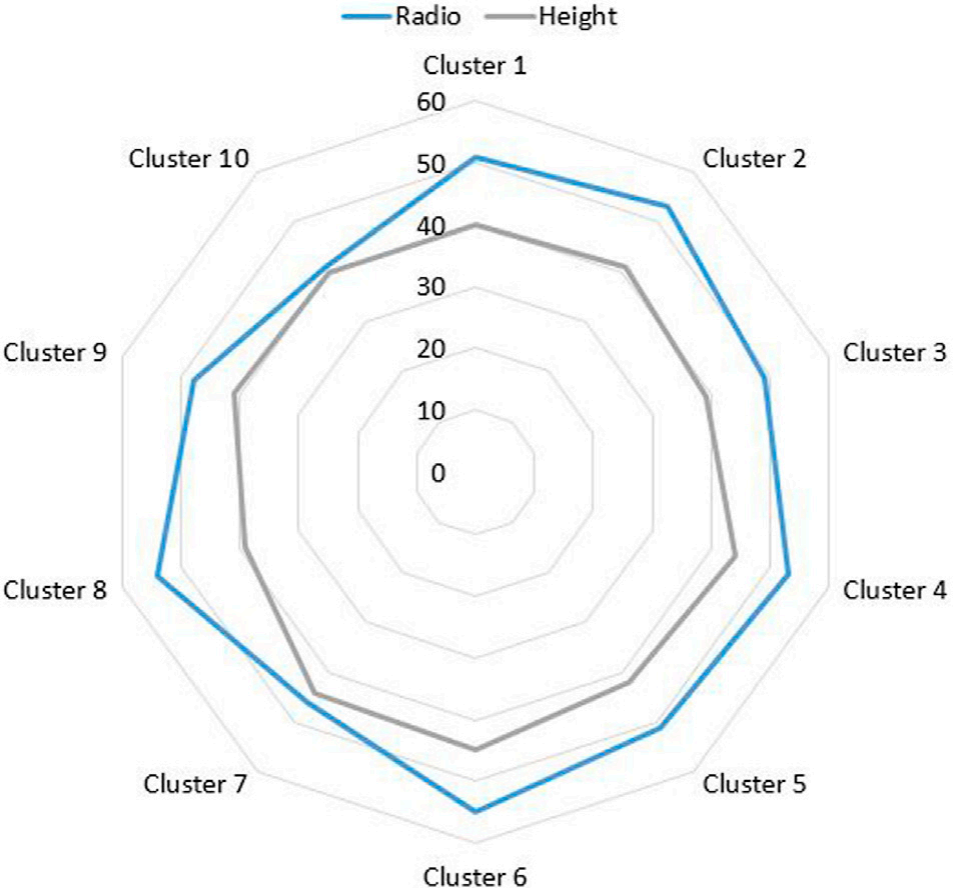
Height and radius dimensions representation of the characterized plants.

Figure 6 shows the representation of the relationship between the radio and heights for each cluster of Figure 5B. These data will later be used to generate the baking trays of the robotic arm. The efficacy of the method has been estimated, obtaining an average efficacy of the 93.1*%*.

A good elimination of outliers and scattered points that can generate conflict in clustering must be previously developed for having a high accuracy index in recognising the number of plants.

### 3.2 Vegetable Identification Using RGB Information

The process developed in this section allows the vegetables to be detected automatically in the RGB mosaic, creating a layer with the vegetables extracted from the crop row. The development of this method is based on the application of histograms extracted from the Green color channel, which provides the most significant amount of information in crop processing applications.

**FIGURE 7:**
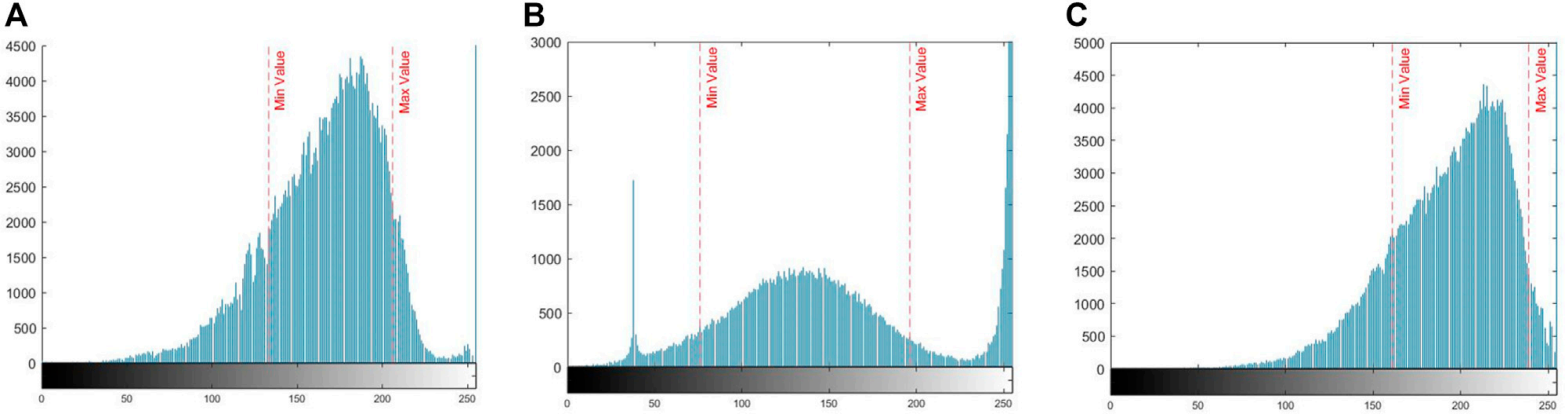
Histograms corresponding to Figure 4A,E,C mosaics, Cabbage, Small cabbage, Red cabbage.

Figures 7A-C shows the histograms of the Green channel, corresponding to the mosaics of Figure 4A,E,C. Two indicators have been automatically placed in the histograms, corresponding to the minimum and maximum values of the threshold values. These values contain approximately 70% of the colour channel relevant information concerning the vegetables in the rows. The rest of the information has been discarded since it corresponds to weeds and noise.

Figure 7B shows two abnormal peaks, which may have their origin in the processing and capturing of the image. The first peak that originates around the value of 40 is probably due to unfiltered noise in the image. However, the second peak may have its origin in the saturation of the green colour channel used, due to a high concentration of pixels with values around 240, in the centre of the plant, which is not a problem for the proposed method since the selective segmentation applied has shown to be functional in the individual plants recognition.

**FIGURE 8:**
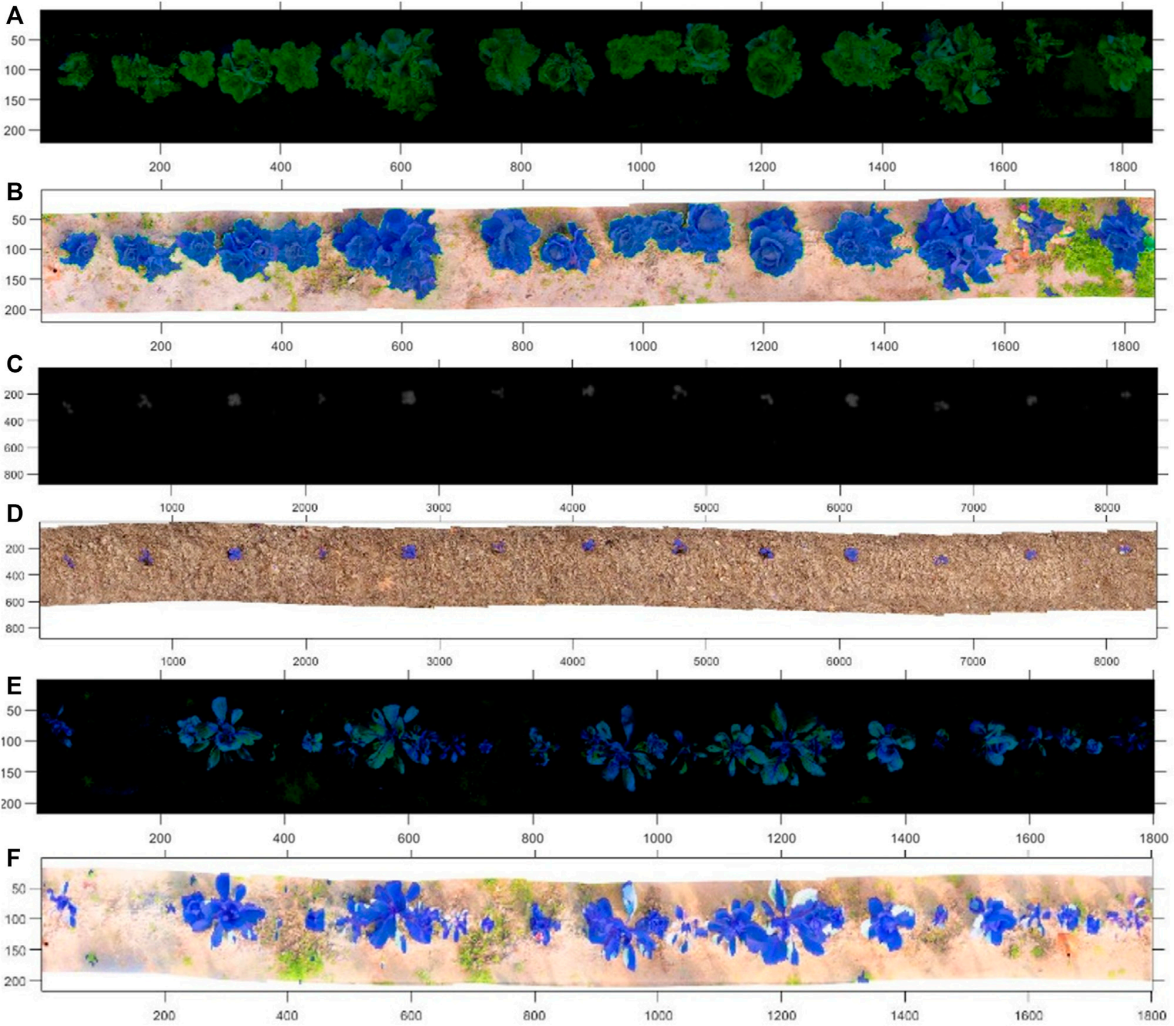
**(A,C,E)** Masks generated from the processing for the detection of vegetables using dynamic histograms. **(B,D,F)** Original images with the mask applied, detected vegetables are highlighted.

Based on these ranges, thresholding is applied. Figure 8A,C,E, show the degenerate layers, which have made it possible to separate the plants from the mosaics. Figure 8B,D,F shows the final identification of the plants superimposed on the original mosaic image. Figure 8B,F, the presence of small weeds can be noticed, which the system has been able to discriminate, segmenting the plants.

The application of histograms has great importance since it allows identifying essential characteristics of the image based on the analysis of histogram pixel concentration areas, which provide relevant information during the detection. The RGB processing method has been evaluated, obtaining an error of 6.4% in detecting vegetables in crop rows.

### 3.3 NDVI Analysis for Decision-Making of Fertilizer Application

One of the main advantages of using NDVI indices lies in the provision of quantitative information on the health status of the plants and the differentiation from the rest of the field. It allows taking actions on specific plants that require fertilization in a crop row.

The detection carried out in the previous section is also used in multispectral mosaics to separate the plants from the rest of the reconstructed scene and later calculate the NDVI indices. To determine those plants that require or do not require fertilizer.

Table 2 shows the mean NDVI indices (x0.01) of the culture rows of Figure 4B,D,F. Based on the definition of the state of the plants [0, −,0.33] Unhealthy plants and [0.33–0.66] Moderately Healthy Plants, it has been possible to determine those that need the application of treatment through the robotic system.

**TABLE 2:** NDVI values (x 0.01) from Figure 4B (Row1)-D (Row2)-F (Row3) are shown. The respective numbering is specified on each floor.

Row	1	2	3	4	5	6	7	8	9	10	11	12	13	14	15	16
1	46	42	4	28	37	29	42	41	46	39	38	27	39	41	–	–
2	43	39	36	23	42	44	26	38	39	41	38	4	29	42	41	–
3	39	24	37	22	4	25	39	27	41	39	3	39	41	42	28	26

The value of the NDVIx0.01 indices of the plants that need the treatment application has been highlighted in red.

In Row 1 and 2, three plants require treatment. However, in Row 3, there is seven small cabbage that requires fertilization. The increase in the number of plants that must be taken care of in this crop row is since the vegetables are in the growth stage and need more nutrients.

### 3.4 Field Fertilization Strategy

The collection and processing of data captured by the three sensory systems proposed in this article have allowed obtaining valuable information on the crop rows, from characteristic-spatial information to vegetative indices, to take corrective actions. The information collection is carried out in a first pass of the platform on the crop row, while in a second pass, action is taken on the vegetables that require it.

The development of robotic fertilization using the Igus CPR5DOF Robotic Arm uses the information processed sequentially in Sections 3.1–3.3. As a basis for trajectory planning.

**FIGURE 9:**
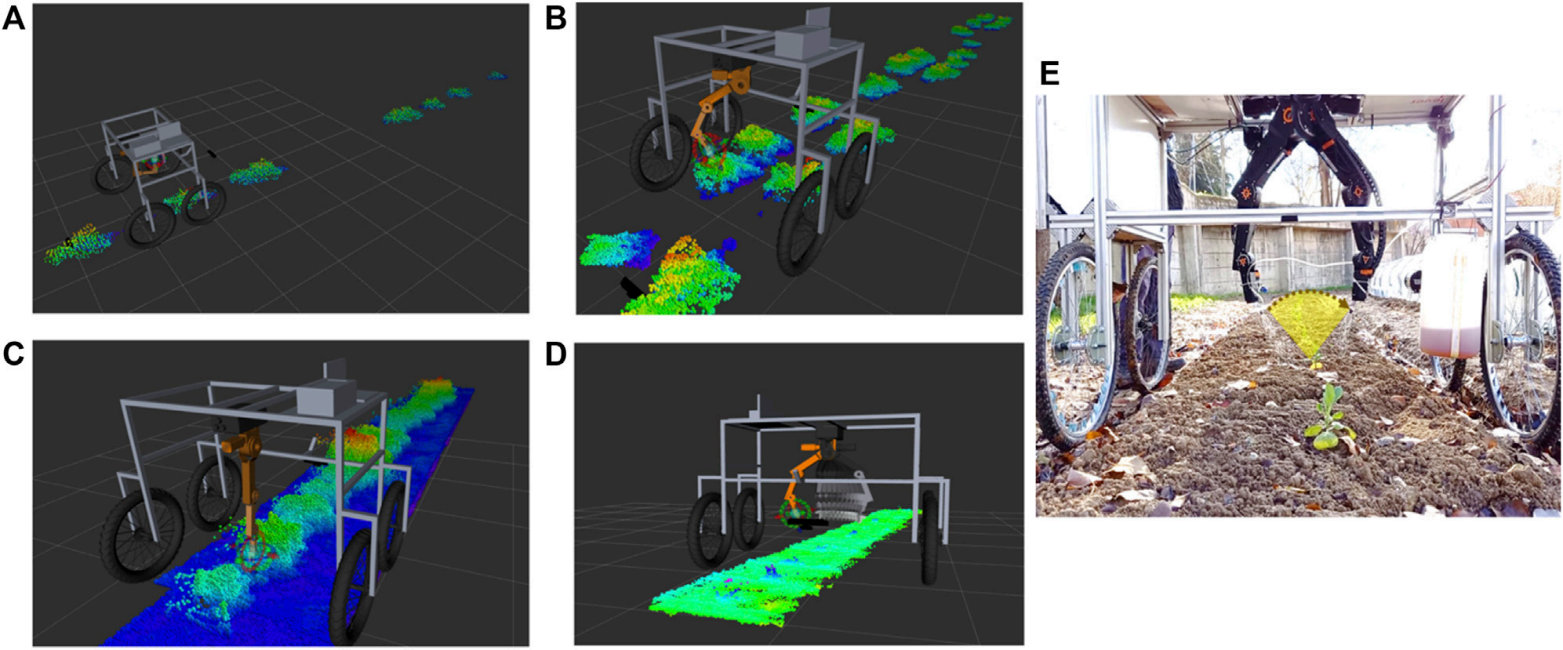
**(A,B,C,D)** Visualization of the perception system and robot movements execution on different rows of crops, using the RVIZ tool for visualization **(E)** Fertilizer application strategies, based on a cone.

In the first instance, the point cloud processing of Section 3.1 allows obtaining the individual clusters and their geometric characteristics to generate a cone that covers all the plant in such a way that the expansion of the fertilizer is homogeneous throughout the plant, as shown in Figure 9E. Subsequently, with the information from Sections 3.2–3.3, it is determined those plants that require the application of fertilizer.

The planning of movements takes as parameters the plant radius, its height and the angle of insertion concerning the base to place the end of the robot in position and perform the sweep, activating the nozzle to apply the liquid fertilizer. The system needs to complete the vision processing analysis and later accomplish its fertilization task in two steps concerning the time processing. The processing of the vision system takes about 200 *ms* due to all the computational processing onboard. On the other hand, the fertilization stage (spraying) takes about 5 s because abundant irrigation with fertilizer is required.

The development of the trajectory planner has been implemented using ROS’s MoveIt tool, which takes as parameters the characteristics and position of the plant, along with the position of the arm to determine the trajectories that the robot will execute. The trajectory that the robotic arm develops is a semicircle at a given height oriented towards the base of the plant, defining a cone that encompasses the plant to fertilize all areas.

The result of the planning executed by the MoveIt package is a discretized collision-free trajectory, which is adapted to the movements of each joint thanks to the controller implemented in python, which takes the current positions, establishes the errors with the destination positions and adjust the speed ramps to obtain a joint movement of the robot. The parameters used for the scheduler are Type of scheduler (RRT - Rapidly Exploring Random Trees), Maximum scheduling time (5 [*s*]), Number of iterations (10), scaling speed (0.4), scaling acceleration (0.3), Goal State (Planning Trajectory Discretized Trajectory), Start State (Current State), Planning Group (Robot CPR Igus-5DOF).

Figure 9A–D shows the robot’s perception system through the RVIZ tool. Different culture rows are shown with the individualized clusters Figure 9-A-B, as well as the robot with the complete culture row Figure 9C,D. Figure 9E shows the nozzle opening of the fertilization system, together with the movement executed by the arm to expand the fertilizer.

### 3.5 Comparison of Sensory Systems for Information Processing

**FIGURE 10:**
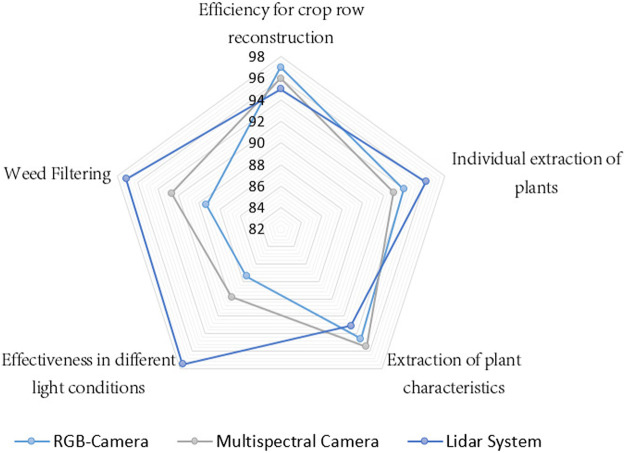
Height and radius dimensions representation of the characterized plants.

Based on the results obtained during the reconstruction of crop rows, processing of acquired data and extraction of relevant information for the robotic fertilization process, the quantitative markers shown in Figure 10 have been extracted, which serve as a reference for analyzing the efficiency of each sensory system used in this experimentation.

The different indicators analyzed in Figure 10 highlight the efficacy of each sensory system (Lidar, RGB, Multispectral). In the first instance, the efficacy for crop row reconstruction has been analyzed, where the three sensory systems have an efficacy superior at 94%, on the other hand, the most robust system for reconstruction in variable light conditions is the laser system, since its operating principle is based on time of flight (ToF). It also has great advantages for eliminating weeds and individual plant characterization. However, the multispectral system is more efficient for the extraction of specific characteristics such as vegetative indices.

It should be noted that each sensory system has greater or lesser efficacy for the silver indicators individually, but as has been shown in this article, the combination of information in a systematic way (Laser System: Reconstruction of rows and geometric characterization of plants, RGB System: Identification of plants, Multispectral System: Obtaining vegetative indices). Allows generating robust processing for the development of a Robotic Fertilization application.

## 4 Conclusion

A method has been presented wich uses three types of sensors (Laser, RGB, Multispectral) to evaluate different parameters in row crops; through its characteristic operating principle, the main conclusions drawn from this work are shown below:

The individual analysis of vegetables in crop rows has a significant advantage over conventional methods in fertilization applications since the proposed method considers specific needs at the plant level to evaluate whether or not the application of treatment is needed. This optimizes the amount of treatment and reduces the potential effect of soil erosion.

Unsupervised learning algorithms allow point cloud processing for plant clustering and the extraction of fundamental characteristic parameters to carry out applications such as robotic trajectory planning.

The use of histograms in the processing of different colour channels (Green) allows identifying areas or regions of interest in the cultivation rows based on the definition of information selection criteria.

The NDVI indices calculated from multispectral images allow evaluating the vegetative state and provide relevant information in making decisions about whether or not to fertilize a plant.

The combined information of the three sensory systems presented has allowed the development of a comprehensive application of a prototype of a robotic platform for fertilization applications. As future research lines, it is proposed to implement a herbicide system to eliminate weeds simultaneously during fertilization on the crop row.

As future lines of research, the analysis of other environmental variables in the fertilization process is proposed, such as the influence of temperature, as well as the use of remote sensors, the development of tests in larger fields is also proposed.

## Data Availability

The original contributions presented in the study are included in the article/Supplementary Material, further inquiries can be directed to the corresponding author.
